# Label-free electrochemical biosensor based on green-synthesized reduced graphene oxide/Fe_3_O_4_/nafion/polyaniline for ultrasensitive detection of SKBR3 cell line of HER2 breast cancer biomarker

**DOI:** 10.1038/s41598-024-62231-8

**Published:** 2024-05-24

**Authors:** Mojtaba Hosseine, Seyed Morteza Naghib, Abbasali Khodadadi

**Affiliations:** 1 School of Chemical Engineering, University of Tehran, P.O. Box 11155/4563, Tehran, Iran; 2 Biomaterials and Tissue Engineering Department, Breast Cancer Research Center, Motamed Cancer Institute, ACECR, Tehran, 1517964311 Iran; 3 Nanotechnology Department, School of Advanced Technologies, Iran University of Science and Technology (IUST), P.O. Box 16846-13114, Tehran, Iran

**Keywords:** Electrochemical immunosensor, Green synthesis, Reduced graphene oxide, Nafion, Fe_3_O_4_, Polyaniline, SKBR3, Label-free detection, Breast cancer, Nanobiotechnology, Electrochemistry

## Abstract

Cancer stands as one of the most impactful illnesses in the modern world, primarily owing to its lethal consequences. The fundamental concern in this context likely stems from delayed diagnoses in patients. Hence, detecting various forms of cancer is imperative. A formidable challenge in cancer research has been the diagnosis and treatment of this disease. Early cancer diagnosis is crucial, as it significantly influences subsequent therapeutic steps. Despite substantial scientific efforts, accurately and swiftly diagnosing cancer remains a formidable challenge. It is well known that the field of cancer diagnosis has effectively included electrochemical approaches. Combining the remarkable selectivity of biosensing components—such as aptamers, antibodies, or nucleic acids—with electrochemical sensor systems has shown positive outcomes. In this study, we adapt a novel electrochemical biosensor for cancer detection. This biosensor, based on a glassy carbon electrode, incorporates a nanocomposite of reduced graphene oxide/Fe_3_O_4_/Nafion/polyaniline. We elucidated the modification process using SEM, TEM, FTIR, RAMAN, VSM, and electrochemical methods. To optimize the experimental conditions and monitor the immobilization processes, electrochemical techniques such as CV, EIS, and SWV were employed. The calibration graph has a linear range of 10^2^–10^6^ cells mL^−1^, with a detection limit of 5 cells mL^−1^.

## Introduction

About 12 million people are expected to lose their lives to cancer by 2030, according to World Health Organization (WHO) projections, making it one of the major causes of death globally^1^. There are presently 439 new instances of cancer (or cancer incidence) for every 100,000 men and women annually. As the most prevalent cause of cancer-related fatalities in 103 countries (lung and cervical cancer ranking first in 43 and 27 nations, respectively), one of the biggest global public health concerns is breast cancer (BC)^2,3^. The high death rate associated with BC is caused by metastasis, an often poor response to treatment, and a lack of early detection. After metastasis, less than 25% of BC patients live for more than five years^4,5^. Proteomics, glycomics, and genome technologies have produced several putative biomarkers that may be used to treat breast cancer. Since the discovery of the first tumor marker in 1847, more than 100 distinct tumor markers have been identified^6^. Due to their presence in blood and ability to convey information about a patient's health, biomarkers have great promise for screening and diagnosis. Gene markers such as BC type (e.g. BRCA1, BRCA2), and protein-based markers such as osteopontin (OPN), cytokeratin 19 fragment (CIFRA-21-1), carcinoembryonic antigen (CEA), human epidermal growth factor receptor 2 (HER2), vascular endothelial growth factor (VEGF), polypeptide antigen (TPA), and cancer antigen CA 27.29, are the most significant biomarkers found in British Columbia^7–9^.

Traditional cancer monitoring methods involved biopsy, which was highly costly, time-consuming, and uncomfortable. Rapid, affordable, highly accurate, sensitive, and selective biosensors for cancer detection were created in response to these issues. The sensitivity and sensing capacity of a biosensor are determined by the materials used to construct a cancer biosensing platform^10,11^. The most commonly used types of biosensors are electrochemical, optical, photoelectrochemical, and piezoelectric biosensors. Electrochemical sensors are the most popular type of sensor on the market due to their inexpensive components, facile fabrication techniques, and affordable sensing equipment^12^. Biomarkers associated with these diseases can be determined ultrasensitively using electrochemical methods such as cyclic voltammetry (CV), chronoamperometry (CA), differential pulse voltammetry (DPV), electrochemical impedance spectroscopy (EIS), and square wave voltammetry (SWV). Applying these methods is simple, inexpensive, reliable, and very sensitive^13^. The use of nanomaterials in the construction of biosensing platforms presents remarkable electrical, mechanical, optical, and magnetic characteristics for these apparatuses. The catalytic activity of sensors can be enhanced by expanding the surface area of the transducing area using nanomaterials. Biosensing applications have made extensive use of electroactive characteristics of nanoparticles toward specific reactions.

The fields of biosensors and electronics have benefited greatly from the utilization of graphene to improve the conductivity, sensitivity, and linear response^14–16^. A sheet of sp^2^-bonded and hexagonally organized carbon atoms found in carbon materials is referred to as graphene which has excellent electrical conductivity^17,18^. Graphene-based nanomaterials have been reported in the literature as biosensors for detecting various analytes, cells, DNA, glucose, and biomarkers^19^. In graphene-based electrochemical sensors, electron transport occurs faster in the vicinity of edges and defects than in the basal planes. Electrochemical sensor applications can benefit from the structural flaws present in chemically modified graphene^20,21^. Graphene's high electrical conductivity and vast theoretical surface area make it an excellent material for electrodes^22^. One of graphene oxide's (GO) most appealing properties is its reducibility to graphene-like nanosheets following the removal of oxygen-containing groups and the restoration of conjugated structures^23,24^. Generally, a reduced graphene oxide (rGO) sheet is called a chemically generated graphene. Graphene-like nanosheets obtained by mechanically exfoliating graphite layers are obtained by a reduction process, and they share common properties and topologies with pure graphene. On the rGO surface, fewer functional groups are visible. Recent interest in rGO has been increased because of its exceptional conductivity, excellent carrier mobility, and large surface area^25,26^. Furthermore, it is a potential biosensing material due to the extremely high stability and ease of functionalization of the reduced chemical groups^27^.

The rGO may be produced by subtracting the oxygen functional groups from GO using compounds such as hydrazine, dimethylhydrazine, hydroquinone, sodium borohydride, hydrohalic acids, and strong alkali^28^. Natural or harmless materials, such as sugar, green tea, ethylene glycol, leaf extracts from natural products, non-aromatic amino acids, and sodium carbonate, have recently been used in the green reduction process^29^. However, owing to the quantity of ascorbic acid (AA) may be adjusted to regulate the degree of reduction, AA has been demonstrated to be an effective reducing agent. The AA, a green chemical, is used in this work to produce rGO.

Magnetite (Fe_3_O_4_) nanoparticles are widely used in the biosensor industry due to their large surface area, high adsorption capacity, good biocompatibility, low toxicity, and ease of manufacture^30,31^. Additionally, Fe_3_O_4_ nanoparticles on the interface of the redox reaction and the electrode surface can greatly speed up the electron transport and improve accessibility to the active surface area^32,33^. Metal oxide nanomaterials typically exhibit greater catalytic activity when it comes to the uniform dispersion of noble metal nanoparticles across a particular surface, as opposed to single-component nanomaterials (like gold nanoparticles). Greater catalytic activity is the outcome of the coordinated action at the metal-oxide support contact^33^.

The semi-flexible conducting polymer known as polyaniline (PANI) belongs to the organic semiconductor family^32^. The utilization of such inexpensive conductive materials has attracted a lot of attention due to their remarkable qualities such as good conductivity, environmental stability, an interesting redox process, and low starting material cost. Polyaniline (PANI), a conjugated polymer, has been proposed for a wide range of applications, including light-emitting diodes, solar cells, biofuel cells, biosensors, supercapacitors, and field-effect transistors. Moreover, PANI and graphene's comparable conjugated π electron structure gives them a significant advantage when creating nanocomposites that combine their best qualities and improve electrochemical performance^34^.

The 185 kDa protein known as HER2, is a member of the receptor tyrosine kinase family. Genes associated with the cell cycle and growth factor receptors (EGFR, FGFR4, and ERBB2) are highly expressed in BC, which may indicate the presence of HER2^35,36^. The fabrication of biosensor platforms that employ different types of graphene to boost the devices' sensitivity and selectivity is described in several papers. Up till now, many research on the fabrication of various electrochemical biosensor types has been published. One such cytosensor developed by Vajhadin et al. can identify HER-2-positive cells. CoFe_2_O_4_@Ag magnetic nanohybrids coupled to HB5 were employed to investigate circulating tumor cells that were CoFe_2_O_4_@Ag positive. These label-free MXene-based cytosensor results showed a low detection limit of 47 cells mL^−1^ and a wide linear range of 10^2^–10^6^ cells mL^−1^^37^. Salahandish et al. created a nanocomposite using silver nanoparticles (AgNPs) adorned with graphene nanostructured PANI to track HER2 BC cells and miRNA-21, which is a good indicator of breast cancer. Excellent reusability in detecting individual cancer cells was made possible by this nanocomposite^38^. A statistical technique called response surface method (RSM) enables the determination of the correlations and interactions between the independent variables and one or more replies. Responses in RSM can be visually represented as counterplots or in a three-dimensional space, which primarily aids in the visualization of the response surface's form^39,40^.

In this work, we take advantage of rGO/Fe_3_O_4_/-Nafion/PANI to fabricate label-free of immunosensors for ultrasensitive diagnosis of HER2. The target is SKBR3, and the biorecognition molecule is the Herceptin antibody. The functionalized electrode was electrochemically tested using CV, SWV, and EIS. The Nafion concentration and incubation time were optimized by us using a central composite design (CCD) and the RSM. With a broad detection range of 10^2^–10^6^ cells mL^−1^ and a low detection limit of 5 cells mL^−1^, the immunosensor was effectively constructed. Additionally, the design process shown here may be used to identify additional signals in clinical diagnostics.

## Experimental

### Materials

The graphite fine powder (spectroscopic grade, particle size ≤ 50 μm), phosphoric acid (85%), sulfuric acid (98%), potassium permanganate (KMnO_4_), hydrochloric acid (36%), AA (C_6_H_8_O_6_), ferrous sulfate heptahydrate, Iron(III) chloride hexahydrate, ammonium hydroxide, Nafion (5% EtOH solution), aluminum oxide, potassium ferricyanide and potassium ferrocyanide were purchased from Merck. N-hydroxysuccinimide (NHS, 98%), 1-Ethyl-3-(3 dimethylaminopropyl) carbodiimide, hydrochloride (EDC, 98%), PBS (phosphate buffered saline), bovine serum albumin (BSA), and aniline (99.5%) were purchased from Sigma-Aldrich. All solutions were prepared using deionized water. Herceptin was obtained from Institute of BioChemistry and Biophysics (IBB, Iran). SK-BR3, MCF7, and LO2 cell lines were obtained from Shahid Beheshti University of Medical Sciences. Glassy carbon electrodes (GCE) were purchased from Azar Elect.

### Methods and apparatus

The morphology characteristics of the nanocomposite were analyzed using transmission electron microscopy (FEI Tecnai F20 series) and scanning electron microscope (AIS 2100, Seron Technologies, Korea) with 5000, 10,000, and 30,000 magnifications. For the ultrasonic irradiation, a PARSONIC 15S ultrasonic bath operating at a frequency of 28 kHz was employed. Every electrochemical experiment was conducted using an Autolab PGSTAT 30 (Echo Chemie, B. V., Netherlands) equipped with a three-electrode apparatus: a modified GCE as the working electrode, Ag/AgCl as the reference electrode, and Pt rod as the counter electrode. To create error bars, each electrode was examined 3 times for each experiment.

### Preparation of nanocomposite electrodes

#### Synthesis of nanocomposite

**Figure 1:**
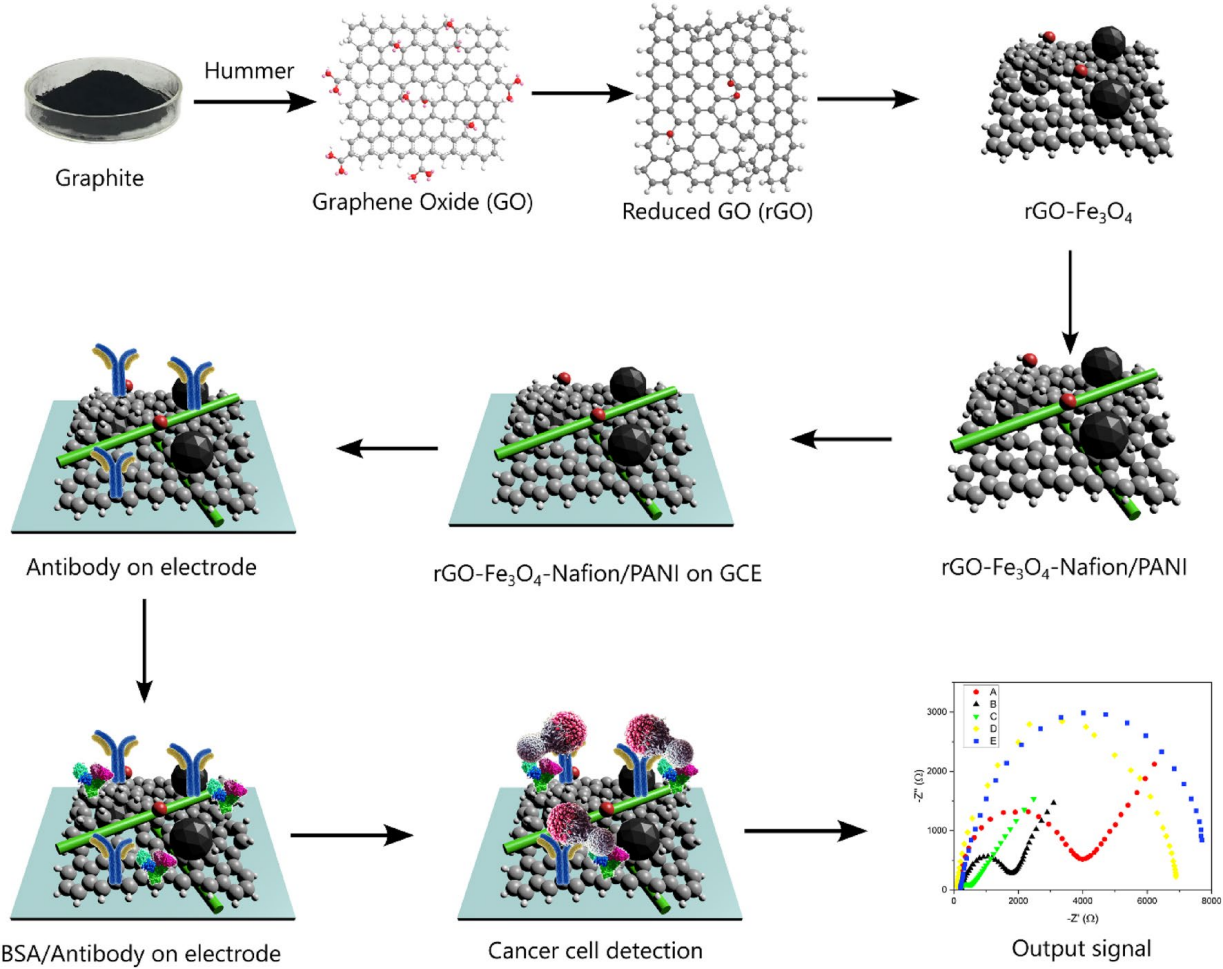
Schematic process diagram for the fabrication and functionalization of sandwich rGO/Fe_3_O_4_/Nafion/PANI for the detection of SK-BR3 cell line.

The process diagram for the creation and functionalization of nano-biosensors employing the sandwich rGO/Fe_3_O_4_/Nafion/PANI for the detection of SK-BR3 is shown in Fig. 1.

#### Graphene oxide synthesis

According to the literature, a modified Hummer was used to generate the GO using graphite fine powder, H_2_SO_4_, H_3_PO_4_, and KMnO_4_. In summary, at 75 °C, a mixture of phosphoric acid, sulfuric acid (40:360 mL), graphite and potassium permanganate (3:18 g) was first formed. After being shaken and stirred for 12 h, the two solutions were cooled to 25 °C and placed in an ice bath with 6 mL of 30% hydrogen peroxide added. Following centrifugation, the final product underwent purification. The resultant dilution was centrifuged, filtered, and washed with a 1:10 chlorhydrate (30% v/v, 250 mL) to remove any remaining ions. The filter was then reused. Before being cleaned with a 200 mL EtOH solution.

### Preparation of Fe_3_O_4_-rGO nanomaterials

#### Green synthesizing rGO

Using 0.1 M AA and 50 mL of GO solution (0.1 mg mL^−1^), a volume ratio of 1:1 was obtained on a heating plate^41^. Following that, the mixture was swirled for 30 min while being heated to 70 °C. The solution's hue shifted from brownish-yellow to black throughout this period. The residual solid (i.e. rGO) was what remained after the product was centrifuged three times with ethanol and water to remove the supernatant. After that, it was baked to dryness at 120 °C.

#### Anchoring Fe_3_O_4_ on rGO

Generally, ultrasonication was used to disperse 500 mg of rGO in distilled water for 2 h. Following the addition of FeSO_4_·7H_2_O (0.002 mol) and FeCl_3_·6H_2_O (0.004 mol), the mixture was ultrasonicated for an hour. About 1.65 mM NH_4_OH solution was dropped and continuously spun for 2 h. Once the resultant black substance was filtered and repeatedly washed with water to remove any residual acid and dissociative Fe(II), it was treated with ethanol. After immersing the solid for 60 min in anhydrous ethanol, then filtered and vacuum-dried at 100 °C^42,43^.

### Optimization of experimental conditions

#### Optimization of Nafion percentages in the rGO-Fe_3_O_4_-Nafion composite

To improve an electrochemical sensor's sensitivity (specific experimental parameters), the ratio of Nafion to Fe_3_O_4_-Nafion in rGO was tuned. By utilizing a CCD and the RSM, we evaluated the impacts of the Nafion concentration ratio and the length of the incubation period. Consequently, Table 2 displays the outcomes of a two-factor and three-level fractional factorial CCD design. The trial's statistical design was created using the Design-Expert 11 tool. We looked at the effects of Nafion percentage in composites ranging from 0.05 to 0.1% w/w and incubation time between 30 and 60 min.

Design of experiment (DoE) evaluated the effects of two factors on current: Nafion percentages and incubation time. Table 1 displays the range of these characteristics that apply. In order to maximize the current, a factorial design was taken into consideration, and Table 2 displays the runs under various circumstances.

**Table 1:** Variables chosen for the experiment's design, as well as their significance levels.

Coded variable	Variable	Level		
		− 1	0	+ 1
X1	Nafion concentration	0.05	0.075	0.1
X2	Incubation time	30	45	60

**Table 2:** Design of experiments: factorial design and supplementary experiments of CCD.

ΔI (µA)	Incubation time (min)	Volume percentage of Nafion (%)
40	30	0.05
58	45	0.05
63	60	0.05
90	30	0.1
102	45	0.1
112	60	0.1
119	60	0.075
125	30	0.075
135	45	0.075
139	45	0.075
140	45	0.075
141	45	0.075
141	60	0.075

#### Modification of GCE and preparation of GCE sensor

Following a thorough polishing process utilizing aluminum oxide 0.05, 0.3, and 1.0 µM powders in that sequence, and a thorough washing in ethanol and water solution between polishing steps, it was ultrasonicated for 5 min, followed by pure water, before being dried with N_2_ gas. 10 µL of the rGO-Fe_3_O_4_-Nafion suspension were poured onto the GCE's surface in the second stage, and it was allowed to air dry. The PANI layer on the electrode surface was then electropolymerized using a solution of 0.2 M aniline and 0.5 M H_2_SO_4_.

As a consequence, the potential range was altered to − 0.15 to 1.2 V and the scan rate was increased to 20 mV s^−1^ for 10 cycles^44^. Microscopic methods like TEM and SEM were employed to verify that the produced nanocomposite and coated electrode met all specifications and was of superior quality. To activate carboxylic groups, the designed electrode was maintained in the (NHS/EDC) dissolution (1.0 mM) for 60 min at room temperature after the rGO-Fe_3_O_4_-Nafion/PANI/GCE was washed with double deionized water (DDW) in the fourth stage. In a wet chamber, 10 µL of a 1 mg mL^−1^ herceptin solution were dropped over the electrode surface for a duration of 12 h, constituting the fifth step. This electrode was designated as rGO-Fe_3_O_4_-Nafion/PANI/GCE. Then, in order to cover any exposed target surface regions that were still uncoated with biomolecules and eliminate nonspecific binding, the rGO-Fe_3_O_4_-Nafion/PANI/GCE electrode was submerged in a 0.1% BSA solution for 40 min.

#### Electrochemical measurements

In an electrochemical cell with 0.1 M PBS (pH 7.4), 0.1 M KCl, and 5.0 mM [K_3_Fe (CN)_6_/K_4_Fe(CN)_6_], CV and EIS measurements were carried out. If CV is scanned at 100 mV s^−1^, it can be attained in the potential range of − 1.0 to 1.0 V. The 10 mV amplitude alternating pulses were used for the EIS investigation. From 10,000 to 0.05 Hz, impedance spectra were recorded. A signal with amplitude of 40 mV and frequency of 60 Hz at the range of 0.5–0.7 V vs. Ag/AgCl reference have been utilized for SWV characterization.

## Results and discussion

### Characterization of rGO-Fe_3_O_4_-nafion/PANI nanocomposites

#### Morphology characterization

On the GC, the prepared material was placed and the electrodes were cleaned accordingly. After that, biosensing selectivity, calibration, and activity tests were utilized. The FTIR spectroscopy of produced materials was assessed to examine the functional groups present in rGO-Fe_3_O_4_ (Fig. 4). Raman spectroscopy is an effective technique for determination of the composition and structural characteristics of materials. Raman spectroscopy was used to investigate the samples in order to determine their structural characteristics (Fig. 5). The VSM test (Fig. 6) was utilized to identify the type of magnetization produced by an applied external magnetic field.

**Figure 2:**
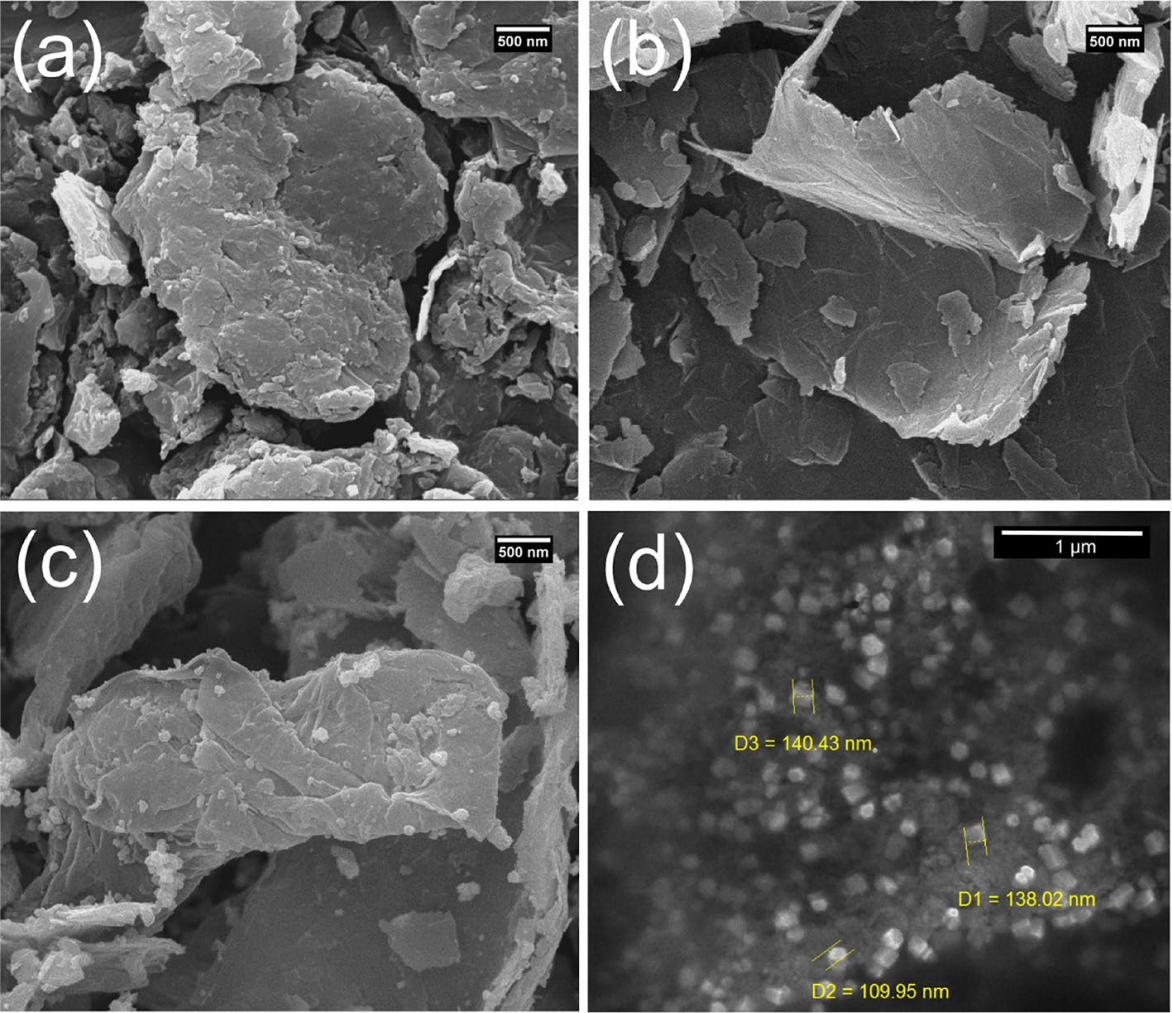
SEM images of (**a**) GO, (**b**) rGO, (**c**) rGO-Fe_3_O_4_, and (**d**) rGO-Fe_3_O_4_-Nafion/PANI.

SEM was used to evaluate the surface morphologies of GO (Fig. 2a), rGO (Fig. 2b), rGO-Fe_3_O_4_ (Fig. 2c), and rGO-Fe_3_O_4_-nafion/PANI (Fig. 2d). From a petal-like angle, the SEM of GO in Fig. 2a depicts the porous graphene nanosheets that resemble nests. Following the reduction procedure, the GO folded sheets showed an exfoliated sheet-like shape and were well reduced, resulting in a greater surface area (Fig. 2b). There are some exfoliated layers and nest-like porosity on the surface of the PANI/GO/GCE electrode, as shown in Fig. 2d. The PANI/GCE coarser's SEM also shows some exfoliated layers. When acid is added to aniline oligomers or aniline amines, a nanowire-shaped PANI is formed, as shown in Fig. 2d.

**Figure 3:**
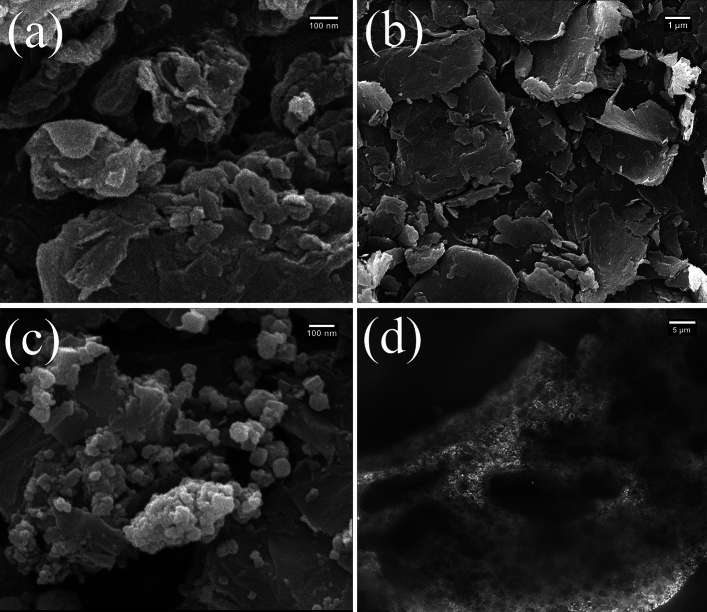
SEM images with different resolutions: (**a**) GO, (**b**) rGO, (**c**) rGO-Fe_3_O_4_, and (**d**) rGO-Fe_3_O_4_-nafion/PANI.

Figure 3 demonstrates the different SEM pictures of the nanocomposites including GO (Fig. 3a), rGO (Fig. 3b), rGO-Fe_3_O_4_ (Fig. 3c), and rGO-Fe_3_O_4_-Nafion/PANI (Fig. 3d). Figure 3a depicts the form of a typical graphene layers. Compared to GO and rGO, Fig. 3a displays the characteristic wrinkled appearance, clumped structure, and sheet-like silky waves. It is evident from Fig. 3c that the Fe_3_O_4_ exhibits homogeneous sphere-like morphologies.

#### FTIR

**Figure 4:**
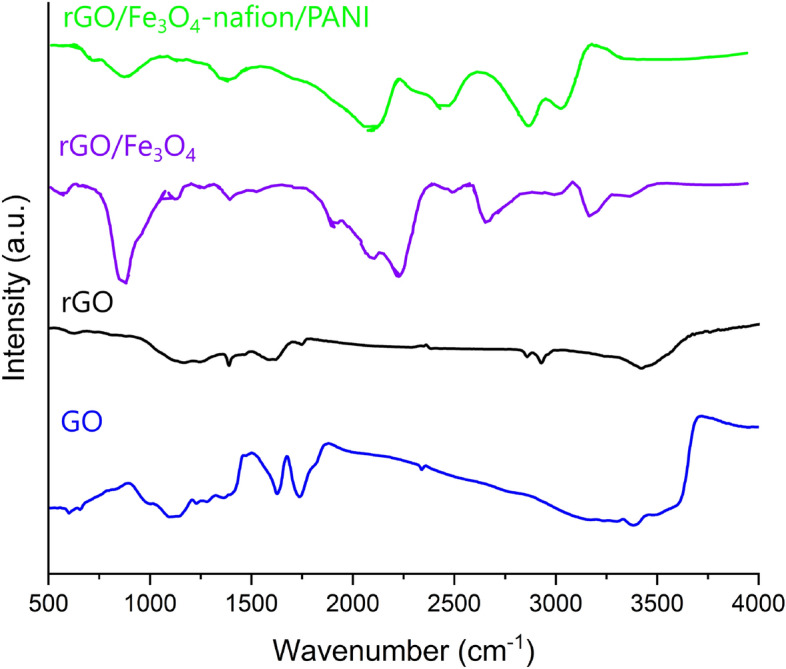
FTIR spectrum of the GO, rGO, rGO-Fe_3_O_4_, and rGO-Fe_3_O_4_-Nafion/PANI.

In Fig. 4, the FTIR spectra of the composites GO, rGO, PANI, and rGO–PANI are shown. The nanocomposite's characteristic FTIR spectrum agrees with earlier research. The characteristic peaks of the GO spectra were formed by C–H stretching, C=C stretching, C–H bending, C–O stretching, and OH out-of-plane bend at 3060.22, 1643.39, 1433.05, 1091.47, and 599.28 cm^−1^, respectively. These peaks were absent from the graphite spectra, indicating that significant oxygen-containing functional groups were added to GO by the oxidation processes^45^.

The rGO peaks, which are 3060.22, 1643.39, 1433.05, 1091.47, and 599.28 cm^−1^, all showed a substantial drop when compared to the GO peak, providing additional strong evidence of reduction. All rGOs exhibit a reduction in the strength of these GO peaks, which suggests a certain degree of elimination of functional groups that contain oxygen^46^.

These functional groups are also present in GO–Fe_3_O_4_, but they are red-shifted in the bond locations and have altered peak sharpness, especially in the case of aromatic C=C bonding. This suggests that different functional groups in GO–Fe_3_O_4_ are operating in a different coordinating context. The peaks in the 400–700 cm^−1^ range correlate to the Fe–O content of Fe_3_O_4_. The suggestion is that covalent bonding in GO is supported by the change in C=C bonding and peak position shift. The decrease of GO is shown by the lower absorbance intensity of several functional groups in rGO–Fe_3_O_4_ as compared to GO–Fe_3_O_4_^47^.

New peaks were also observed for the C=N (1571 cm^−1^), C–N (1237 cm^−1^), C–H stretching vibration (873 cm^−1^), and C=C of the quinoid and benzenoid rings (1486 and 1300 cm^−1^) of PANI. The successful integration of PANI was confirmed by these outcomes^48^.

#### RAMAN

**Figure 5:**
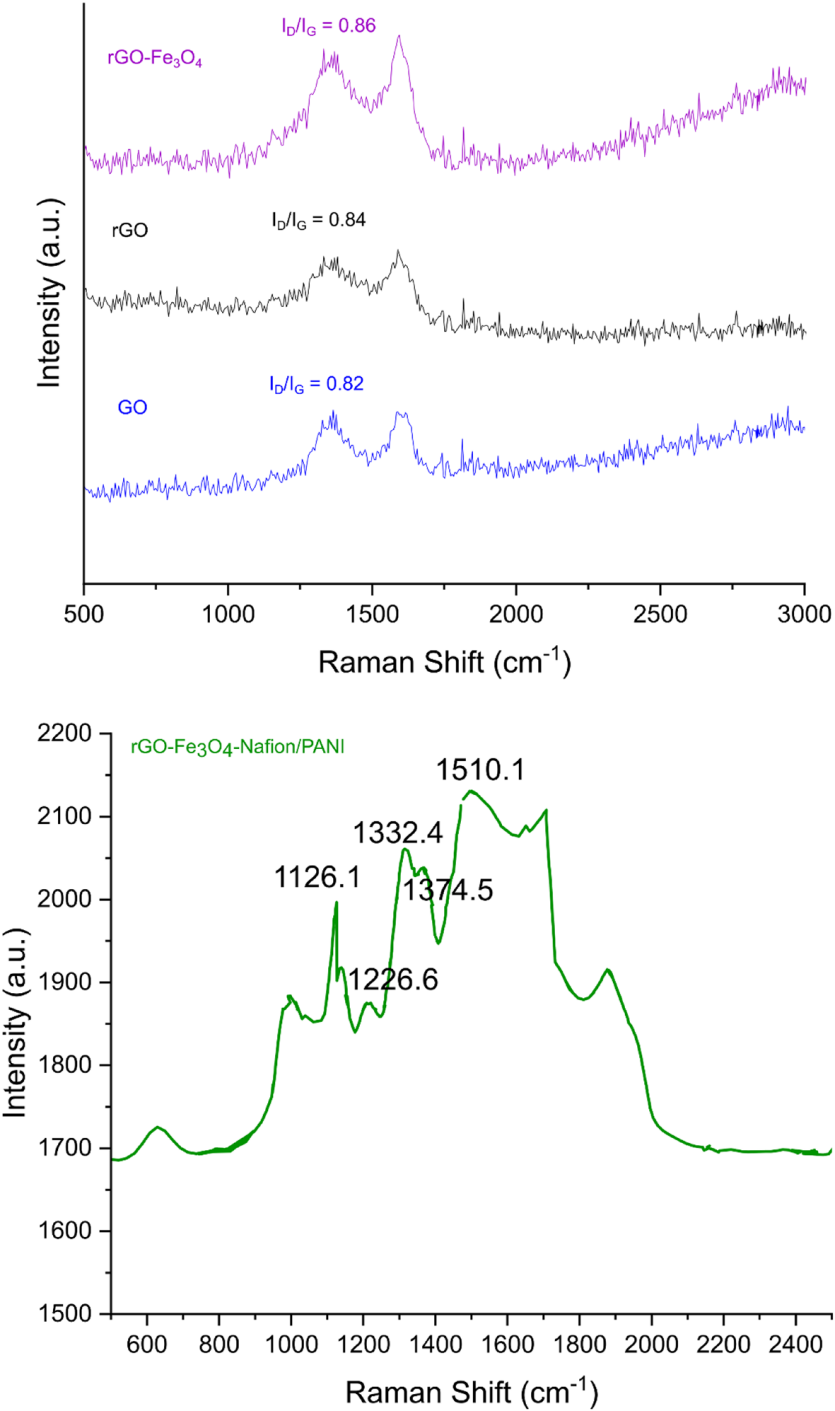
Raman spectra obtained for GO, rGO, rGO-Fe_3_O_4_, and rGO-Fe_3_O_4_-Nafion/PANI.

Raman spectroscopy is the most efficient technique for characterizing carbon-based materials. The Raman spectra of GO, rGO, rGO-Fe_3_O_4_, and rGO-Fe_3_O_4_-Nafion/PANI nanocomposites are shown in Fig. 5. The breathing mode of j-point photons with A1g symmetry produces the D vibration band, which may be seen at 1364 and 1354 cm^−1^. Figure 5 shows that it is 20 cm^−1^ for rGO and GO, respectively. On the other hand, the G vibration band was found to be produced at 1594 cm^−1^ for GO and 1600 cm^−1^ for rGO by first-order E2g phonon scattering from sp^2^ carbon. All sp^2^ carbon systems have a stretching C–C bond, which also affects the G vibration band. The disorder bands and tangential bands are represented, respectively, by the Raman spectra's D and G bands.

It was found that GO has an I_D_/I_G_ ratio of 0.855. Because the sp^2^ carbon was restored after reduction, the average sizes of the sp^2^ domains decreased while the ID/IG for rGO rose. The greater intensity in the D band was another clue that rGO included more isolated graphene domains than GO; this difference might have been caused by the O_2_ moiety in GO being removed during reduction. It was discovered that GO's ID/IG ratio was 0.855. The average sizes of sp^2^ domains shrank and the I_D_/I_G_ for rGO increased upon restoration of sp^2^ carbon following reduction. The greater intensity in the D band, which was most likely brought on by the removal of GO's oxygen moiety during reduction, provided additional proof that rGO contained more isolated graphene domains than GO^49^.

The peaks that appeared in the ternary composite's Raman spectrum reveal PANI chains that developed on the surface of the rGO/Fe_3_O_4_ composite. These peaks combine the bonds seen in the PANI and rGO/Fe_3_O_4_ samples. Because of the π–π interaction between PANI and rGO, the G band blue-shifts in the Raman spectra of rGO/Fe_3_O_4_/PANI while the D band remains unchanged^50,51^. It was indicated that PANI had been added to the composites by the existence of peaks in the rGO/Fe_3_O_4_/PANI spectrum. The C–C stretching (1397 cm^−1^), C=C (1483 cm^−1^), and in-plane C–H bending of the benzenoid ring (1250 cm^−1^) of the quinoid ring were all correlated with these peaks.

### Magnetic properties

**Figure 6:**
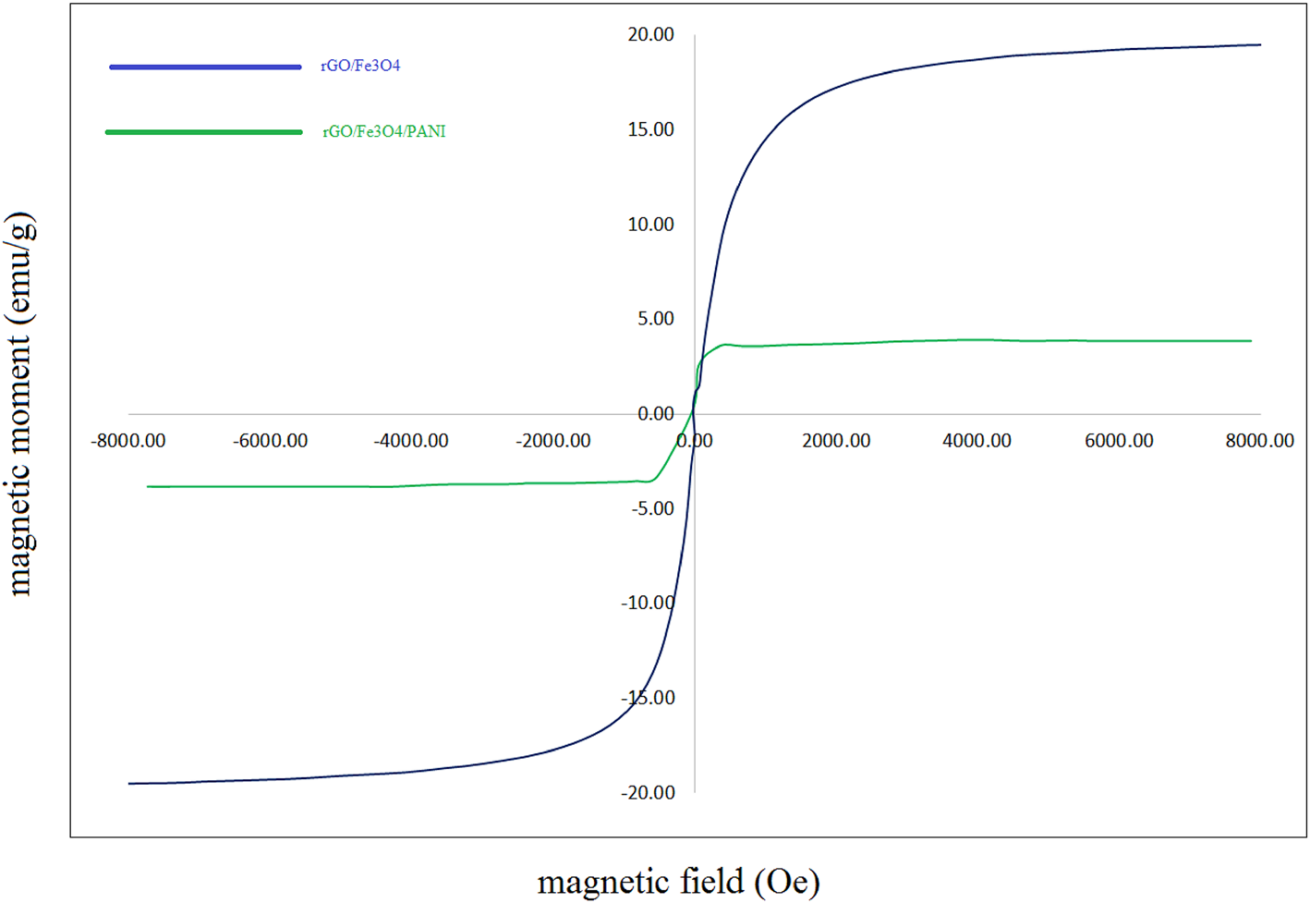
The result of the VSM samples, including rGO-Fe_3_O_4_, and rGO-Fe_3_O_4_-Nafion/PANI.

In the magnetic field range of − 8000 to + 8000 Oe, the M–H curves of the room-temperature rGO/Fe_3_O_4_ and rGO/Fe_3_O_4_/PANI composites are displayed in Fig. 6. The VSM investigations of the GO and rGO (nanocomposite) show that both graphs have very low magnetic remanence. This suggests that when the external magnetic field is removed, the magnetic property disappears^52^. Hence, rGO-Fe_3_O_4_ and rGO/Fe_3_O_4_/PANI show superparamagnetic characteristics. Since rGO-Fe_3_O_4_ has a coating of PANI covering its surface, indicating that the rGO/Fe_3_O_4_/PANI nanocomposite was successfully created, the rGO/Fe_3_O_4_/PANI nanocomposite's SM value is lower than rGO-Fe_3_O_4_^53^.

### Electrochemical characteristics

**Figure 7:**
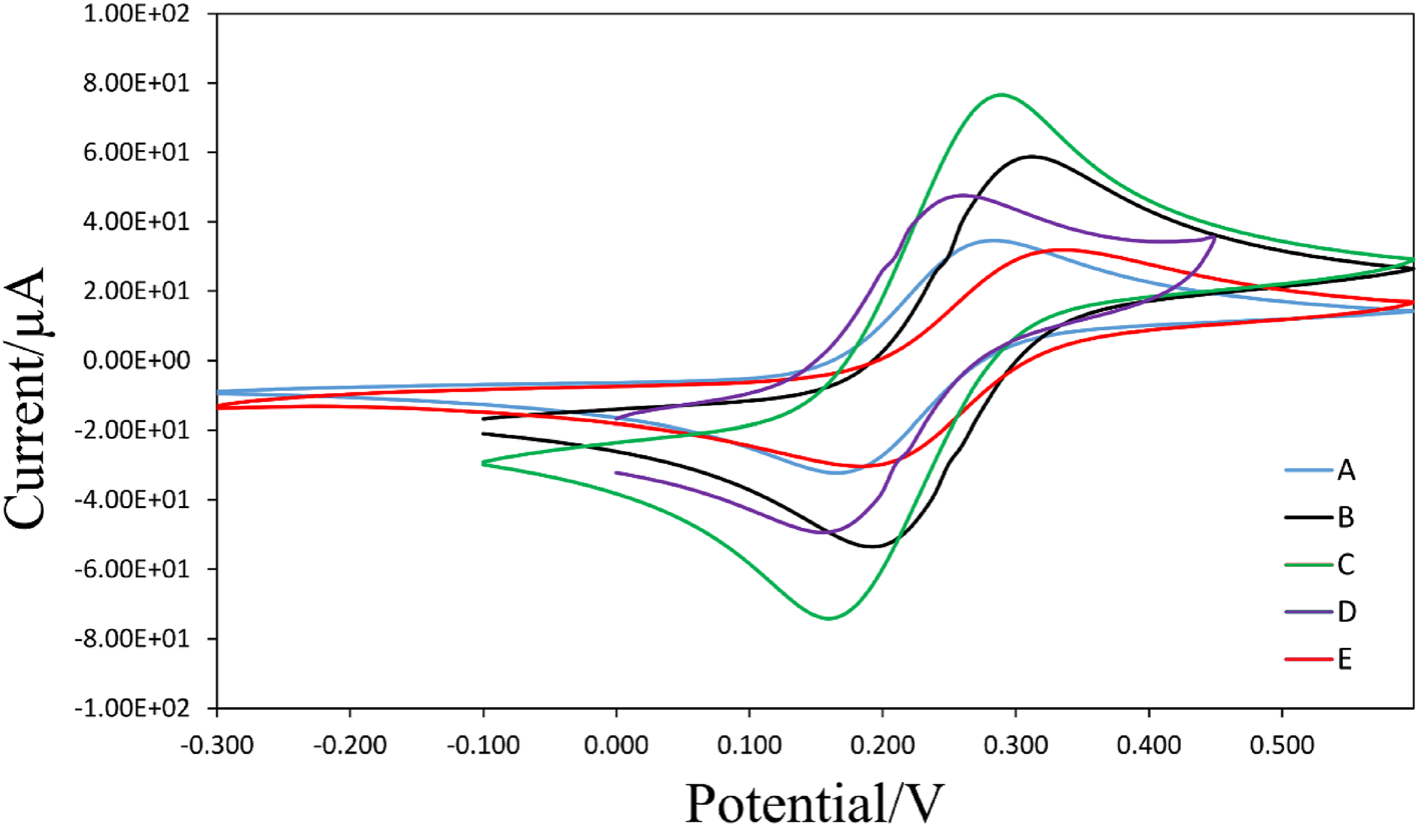
Cyclic voltammograms (CVs) for the bare electrode (A), rGO/Fe_3_O_4_-Nafion (B), rGO/Fe_3_O_4_-Nafion/PANI (C), rGO/Fe_3_O_4_-Nafion/PANI/Herceptin (D), and rGO/Fe_3_O_4_-Nafion/PANI/Herceptin/cells (E), respectively.

**Figure 8:**
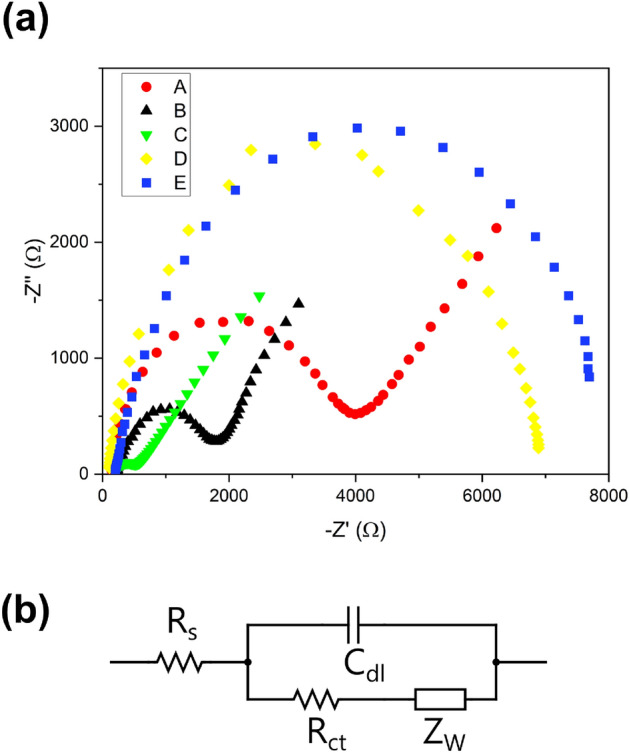
(**a**) EIS for the bare electrode (A), rGO/Fe_3_O_4_-Nafion (B), rGO/Fe_3_O_4_-Nafion/PANI (C), rGO/Fe_3_O_4_-Nafion/PANI/Herceptin (D), rGO/Fe_3_O_4_-Nafion/PANI/heceptin/cells (E) and (**b**) equivalent circuit model of the EIS.

The Nyquist plot and CV are clearly understood, as shown in Figs. 7 and 8, with the peak height of the CV lowering when the electrode was functionalized using rGO-Fe_3_O_4_-Nafion/PANI/GCE. The rGO-Fe_3_O_4_-Nafion/PANI and glassy carbon electrode's effective charge mediation may be the reason why the redox peaks of the rGO-Fe_3_O_4_-Nafion/PANI/GCE with cells were lower than those of the bare electrode. One method used in electrochemical characterization is impedance spectroscopy. Nevertheless, the impedance approach is only partially quantitatively applied for biosensing assessment and detection. Moreover, interferences may affect the results and reduce selectivity as this method uses resistance to the analyte's detection.

#### CV

After the electrode was modified with a nanocomposite, BSA, and cancer cell in a Fe(CN)_6_^3−/4−^ probe, CV was achieved to evaluate electrochemically the surface change of the electrode (Fig. 7). For bare GCE, a very low background current was seen (Fig. 7a). Nevertheless, when standard rGO/Fe_3_O_4_-Nafion was adapted onto the electrode, two oxidation and reduction peaks were seen, and the voltammetric signal rose. Figure 7c shows that the reversible electron transfer process benefited from the presence of rGO/Fe_3_O_4_-Nafion. Comparatively speaking, the modified electrode increased the effective surface area, raised the electron transfer rate, and raised the electrochemical activity. Peak current is likely higher because of rGO's special qualities, which include large surface area, fast electric transfer rates, and superior conductivity. Moreover, using Fe_3_O_4_ nanoparticles can boost the electrode's accessible surface area.

The inset of Fig. 7 illustrates how the peak current rises and how the CV response of rGO/Fe_3_O_4_-Nafion is notably lower than the CV response. An enhancement in the electrode's conductivity, a reduction in the absence of PANI, and an increase in the active surface area all contribute to these outcomes. Stated differently, the PANI electrodeposition led to an increase in the cover's electrode performance and activity. As anticipated, Herceptin immobilization results in a sharp drop in current, which is further diminished when cancer cells are added. These results show that the addition of Herceptin to the surface electrode was successful. Throughout the building process, the surface changes were tracked using the CV.

#### EIS

Due to rGO's high conductivity and wide surface area, as shown in Fig. 8, adding Fe_3_O_4_-rGO to a bare electrode increases the electrode's surface area and lowers charge transfer resistance (R_ct_) when compared to bare GCE.

Following the application of the rGO/Fe_3_O_4_-Nafion-PANI nanocomposite to the GCE, the bare GCE's R_ct_ was significantly reduced. The resistance drops greatly increased the electrode's sensitivity and detection limit. Due to the non-electroactive biomolecule of the antibody, which blocked the surface and impeded charge transfer after it was deposited, the antibody's attachment to the nanocomposite's surface greatly reduced the R_ct_. The R_ct_ increased when BSA was introduced after that. The incubation time resulted in specific adsorption between the antibody and antigen, which preferentially adhered the cancer cells to the functionalized electrode and significantly raised R_ct_.

#### Optimization of the Herceptin concentration and incubation times

The Herceptin concentration was further adjusted for several probe concentrations of 1 × 10^–8^, 2 × 10^–8^, 5 × 10^–8^, and 1 × 10^–7^ M. To enhance this characteristic, several incubation times, including 30, 40, 60, and 120 min, were explored. The findings show that the ideal duration of contact is around 30 min, during which time all necessary incubation is finished. To enhance the biosensor's detecting capabilities, it is imperative to augment the biomolecule's immobilization amount.

#### Optimization of temperature and pH

**Figure 9:**
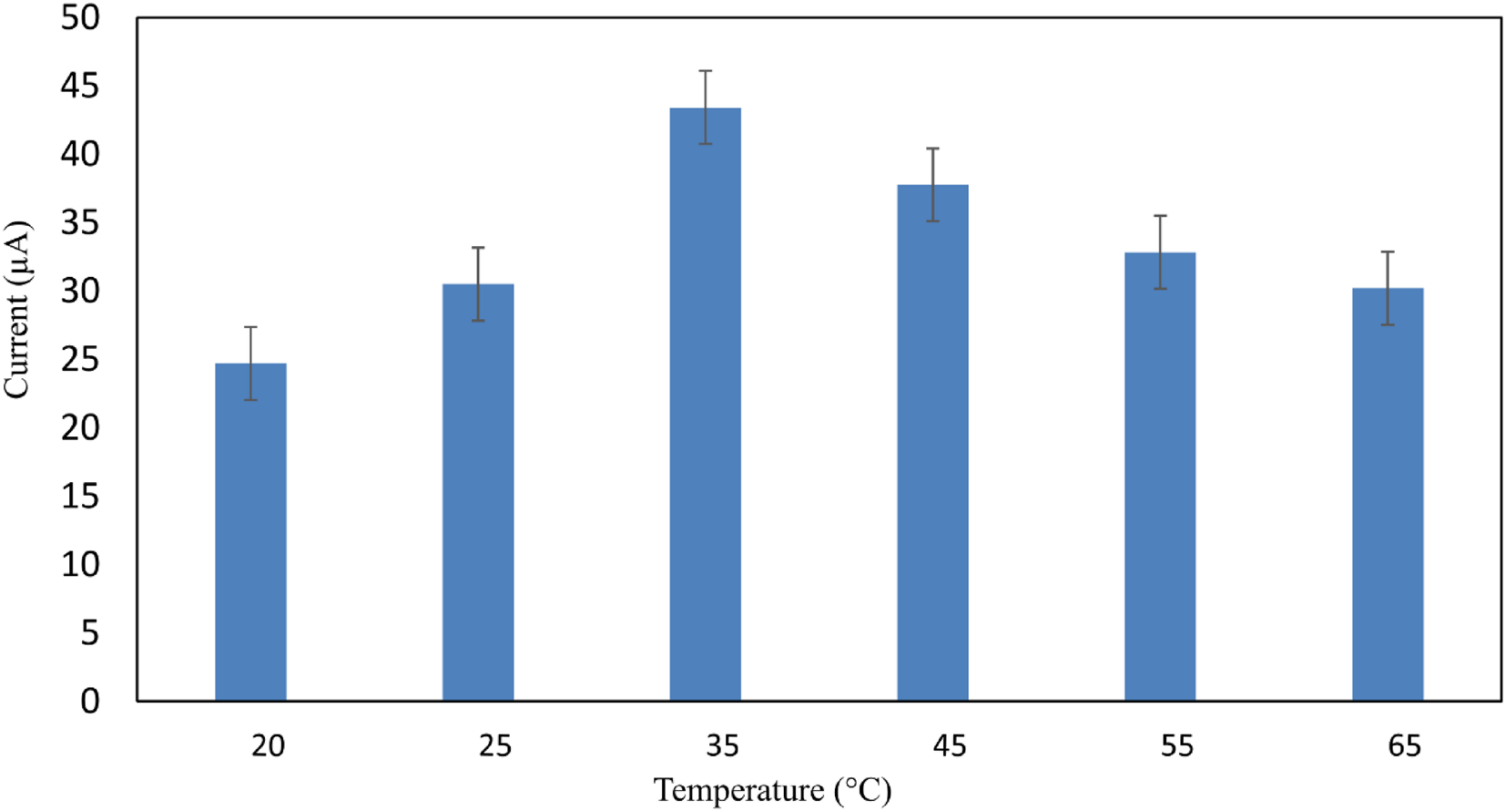
Optimization of temperature in a solution containing 0.5 mM [Fe(CN)_6_]^3−/4−^ in a 0.1 M KCl (n = 3).

**Figure 10:**
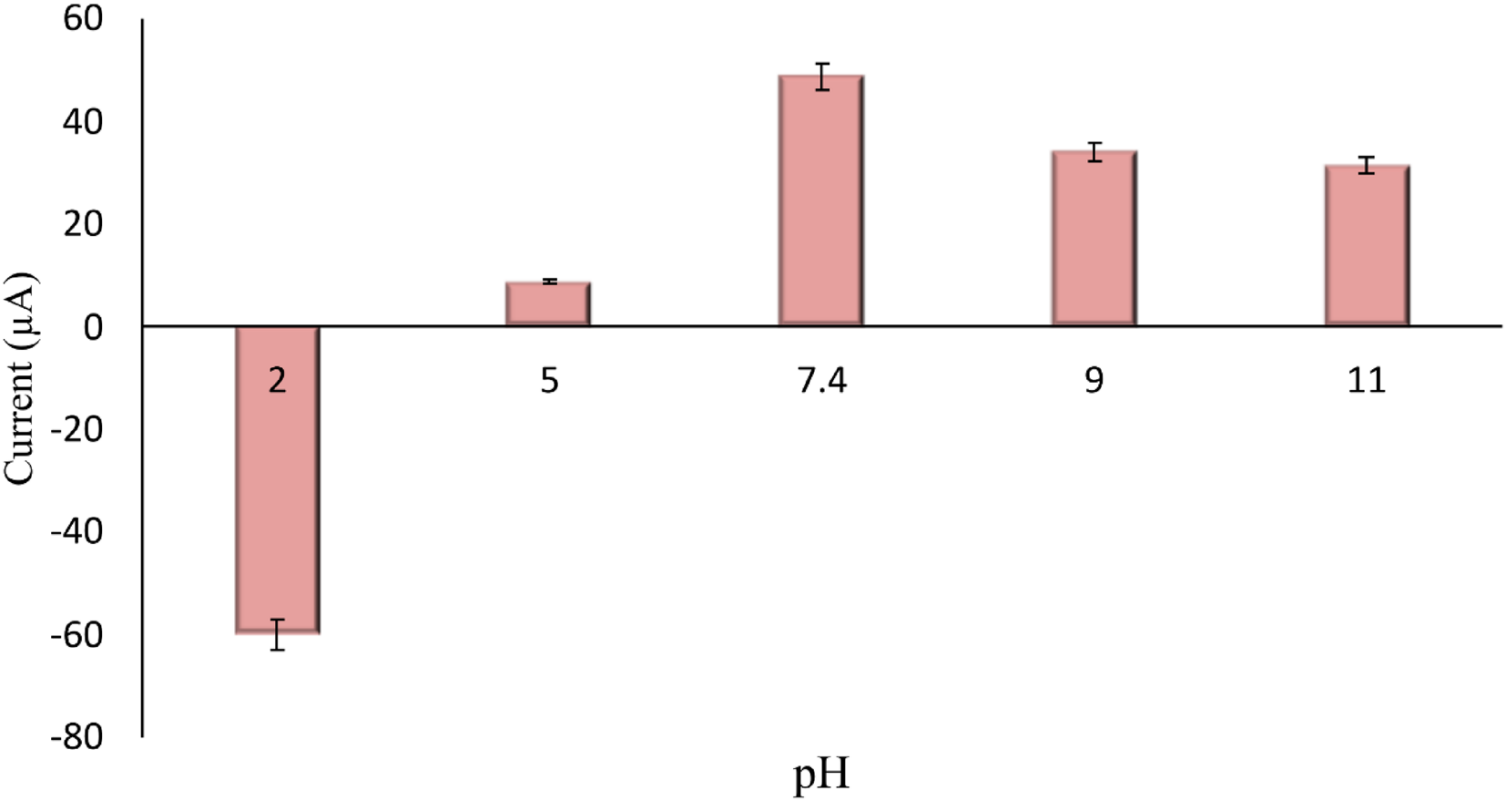
Optimization of pH in a solution containing 0.5 mM [Fe(CN)_6_]^3−/4−^ in a 0.1 M KCl (n = 3).

The electrolyte's temperature affects the electrochemical reaction. Consequently, the nano-performance biosensor was evaluated across a range of temperatures (30, 35, 45, 55, and 65 °C). Because biomolecules are heat-sensitive by nature, it was shown that 35 °C, or almost human physiological temperature, was the ideal working temperature for biomolecule detection in Fe(CN)_6_^3−/4−^ solution (Fig. 9). Not to mention, the pH level needs to be changed. Biomolecules can break down into their parts, such as sugar, bases, and phosphate, in an extremely acidic incubation medium, but they can also be disturbed in a somewhat acidic environment. Hydrogen bonds maintain the interaction between the antigen and the antibody. Furthermore, the hydrogen bonds between antibody pairs may cause the antigen's structure to be disturbed. To investigate how pH affected the biosensors' stability, a variety of pH-varying electrolytes were found (Fig. 10). It has been demonstrated that a pH of 7.4 produces the best biosensing system. After adjusting the functionalization settings, the best values—including sensor calibration—are chosen for the biosensing study.

### Experimental design

The data was analyzed using five different models: mean, linear, 2FI, quadratic, and cubic. The quadratic model was selected due to its highest-order polynomial and substantial sum of squares. The response surface quadratic model's Analysis of Variant (ANOVA) was used to determine the parameters influencing cell growth and the fitted model's significant level. The quadratic model's ANOVA is displayed in Table 3.

**Table 3:** ANOVA for response surface quadratic model.

Source	Sum of squares	Degrees of freedom	F value	*p* value
Model	14,396.53	5	46.83	≥ 0.001
A (time)	253.50	1	4.12	0.0819
B (nafion)	3408.17	1	55.43	0.0001
AB	0.2500	1	0.0041	0.9509
A^2^	316.62	1	5.15	0.0575
B^2^	7672.62		124.79	≥ 0.0001

The F and *p* values show the intensity of the interaction between each independent variable and the importance of each coefficient. The model is significant if the value of "Prob > F" is less than 0.005, and vice versa. Table 3 indicates that the model's p-value was less than 0.001, and its F-value was high, suggesting that the model was important in explaining the data. This suggested that the model provided the necessary signals for navigating the design space. An excellent indicator was the R^2^ value of 0.9710, which demonstrated that a significant amount of variability—up to 98%—was explained by the data.

**Figure 11:**
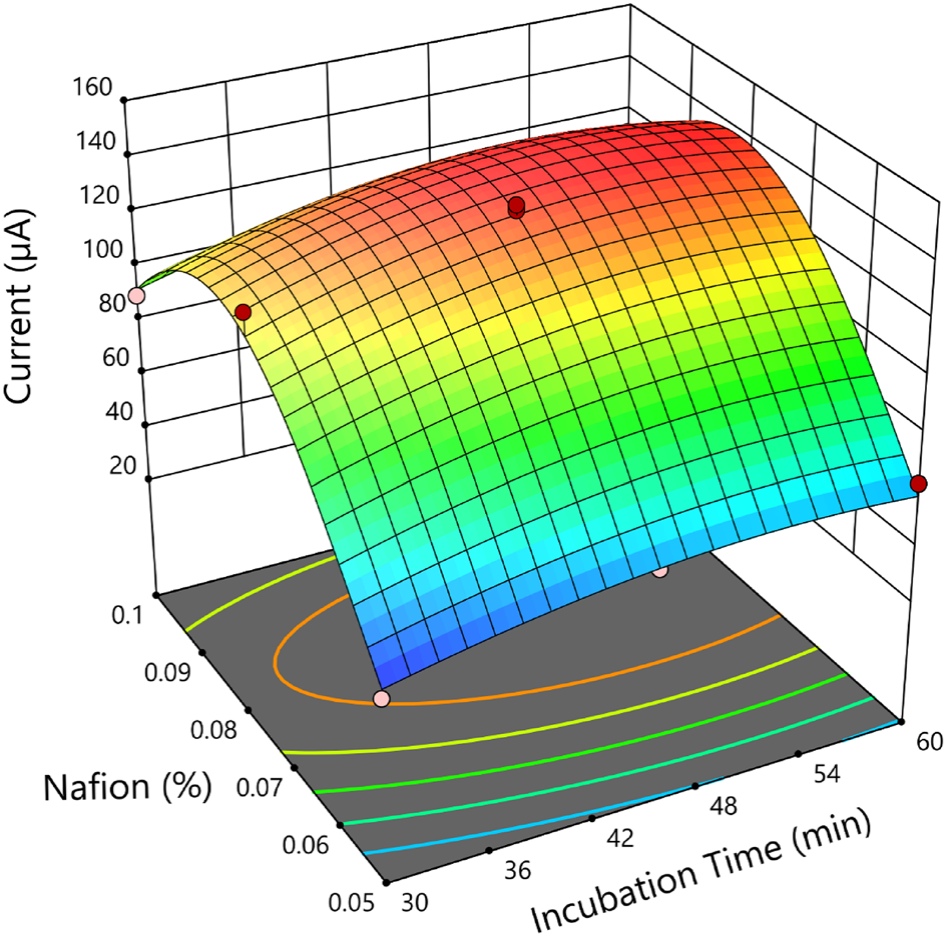
A 3D response surface of interaction between incubation time and nafion.

All two of the parameters had a statistically significant favorable influence on the development of electrical current, according to the ANOVA analysis. The coefficients found in the final equation, according to Anderson and Whitcomb, may be directly compared to determine the relative importance of the various elements. Figure 11 utilizes a bar graph to demonstrate the relative influence of the influencing elements. This figure demonstrated how each factor's influence on each response changed, leading to the conclusion that agitation speed had the most effect on current.

Consequently, a quadratic polynomial equation was used to regress the experimental results derived from the CCD. The resulting regression equation is displayed below, with the actual elements being represented.

$${\text{Current}} = {137}.{34 } + { 6}.{\text{5A }} + { 23}.{83} {\text{B}}^{{2}} - {25} {\text{AB }} - {1}0.{71} {\text{A}}^{{2}} - {52}.{71} {\text{B}}^{{2}}$$

Using software designed to maximize current at a single ideal state, a numerical optimization process produced all the optimum circumstances. The optimum response was validated using a nafion concentration of 0.081% and an incubation duration of 49.77 min. The following graphic illustrates, in three dimensions, how the two critical factors—nafion % and incubation time—affect the reaction to the flow differential. In the accompanying image, the Design Expert program offers the ideal circumstances.

**Figure 12:**
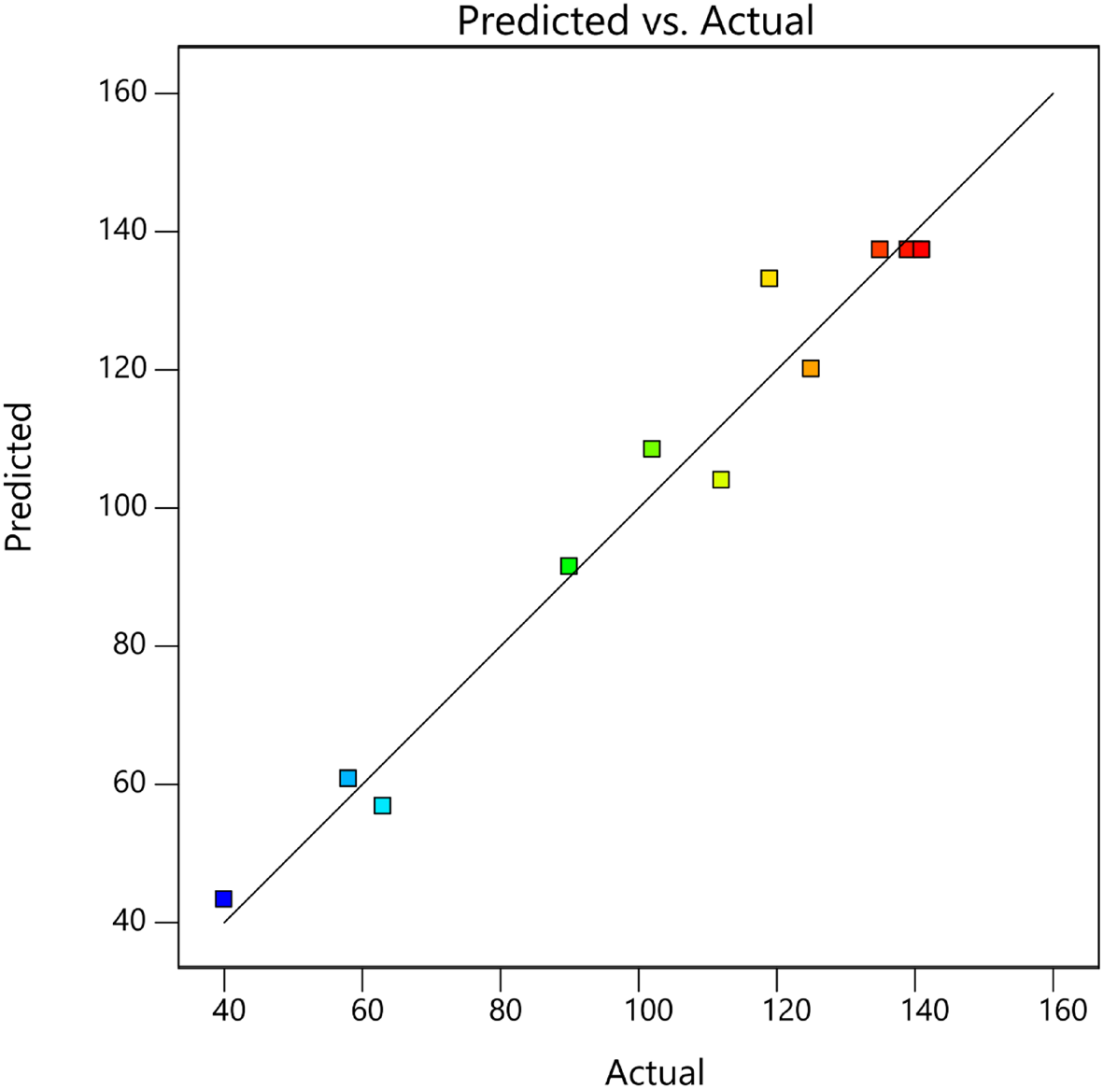
Predicted vs. actual values based on CCD.

Figure 12 compares the actual and expected values. It was successful in bridging the association between process variables and the current that the projected values achieved.

## Nano-biosensor sensitivity, selectivity, stability

### Selectivity analysis

**Figure 13:**
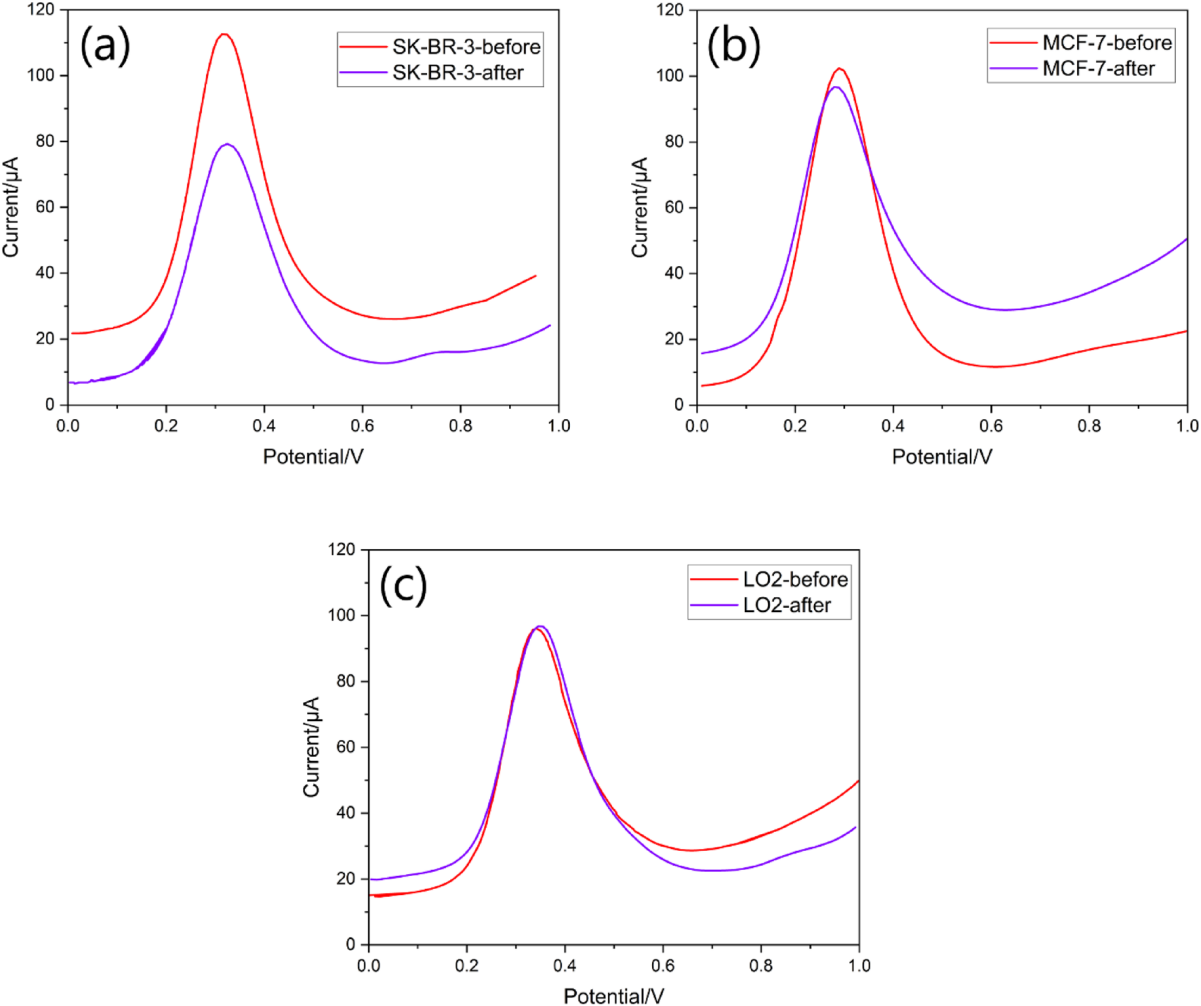
Selectivity of the biosensor and the comparison of different cell lines on the sensing performance (**a**) SK-BR-3, (**b**) MCF-7, and (**c**) LO2. Red and violet curves are related to before and after depositing cells, respectively.

**Figure 14:**
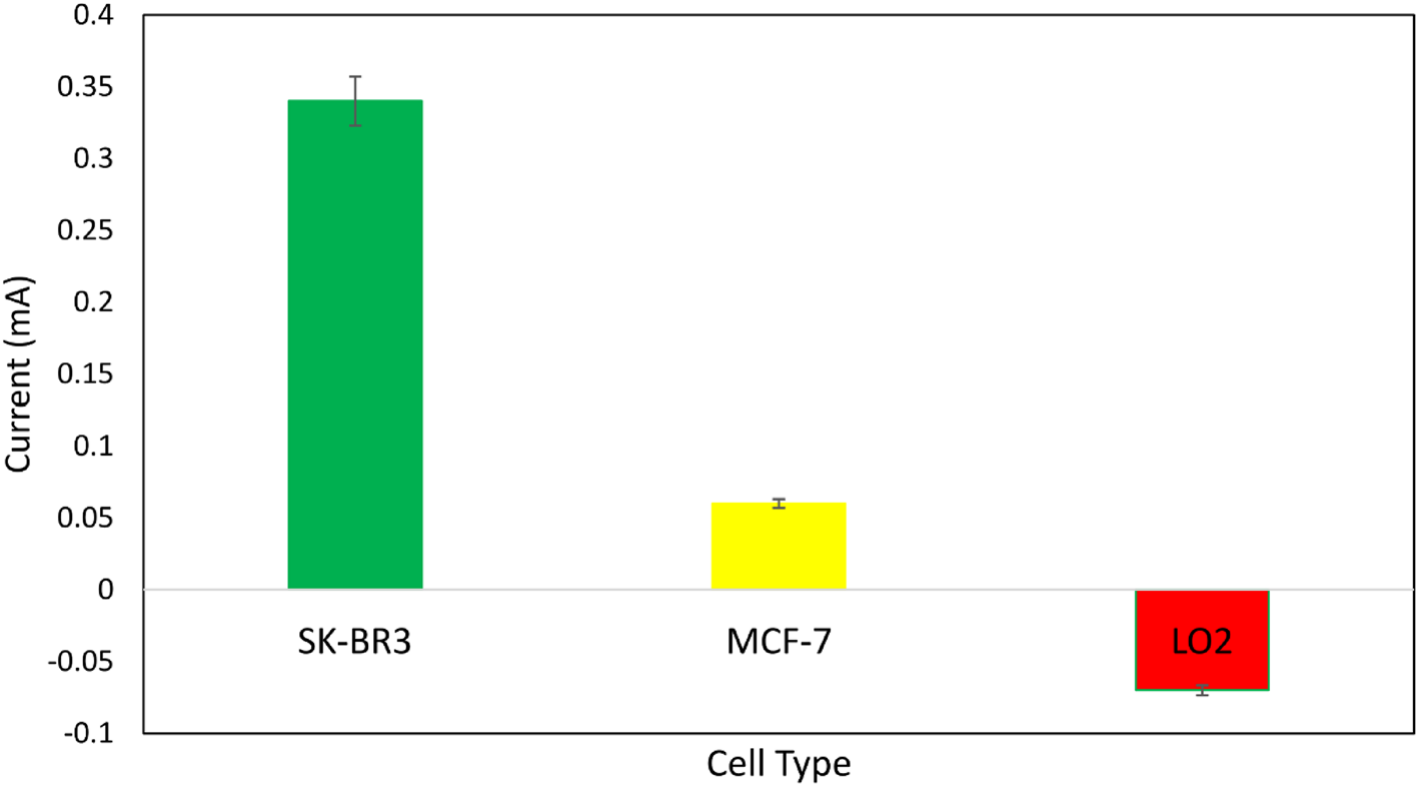
Effect of several cells on the electrode response and a comparison of the biosensor activity in the presence of the cells of MCF-7, SK-BR-3, and LO2.

The most crucial components of a biosensor's sensing ability are its selectivity and responsiveness in real biospecimens containing electroactive chemicals. For this investigation, we used a range of cell lines, including the human normal liver cell line, MCF-7, and SK-BR-3. At each step, 100 cells mL^−1^ of cell solution were drop-coated. SWV analysis was performed prior to and after insertion of the cells onto the electrode surface in each case. We determine the parameter ΔI/I by dividing the intensity difference between SWV peaks on the main intensity (prior to cell implantation) in order to exclude the electrode difference parameter from the selectivity findings. Figures 13 and 14 display the whole data comparison together with the pertinent curves. Compared to the findings for the other cell lines, SK-BR-3 cells appear to have a ΔI/I ratio greater than 0.34. This suggests that in SK-BR-3 cells, the number of cells linked to electrodes was greater than that of other cells, indicating the exceptional affinity and selectivity of Herceptin antibodies for the HER2 receptors. Figure 13 displays the relevant curves together with the results of the data comparison.

The linear range of biosensor typically indicates the range of analyte concentrations for which it displays an acceptable linear corresponding response. To accomplish the linear dynamic range of immunosensor for detecting HER2 + over-expression on the SK-BR3, an updated electrochemical cell and rGO-Fe_3_O_4_-Nafion/PANI electrode were utilized. By adding SK-BR-3 to the electrode surface in varying quantities, the biosensor's linear range was determined. After that, the SWV graphs were compared, leading to the following deductions.

We found that for 4 out of 6 tests, the ΔI/I was greater than 0.34. We also tested the practicality and durability of the synthetic material for 2 weeks. So, not only the hybrid material could be made quickly and cost-effectively, but this biosensor could also be used to detect samples and improve performance. As shown in Fig. 14, the negative value of LO2 is related to the lower value of the before curve (red) in comparison to the after curve (violet) of LO2 in Fig. 13. The LO2 cell line have been utilized as healthy cell measurements where the Herceptin and electrode couldn’t detect it as the affinity of their binding site is lower. Thus, in binding or engagement cases, the cell presence increases the total resistance/impedance of the sensor. It may be related to the electronegativity of the LO2 cell line.

### Sensitivity

**Figure 15:**
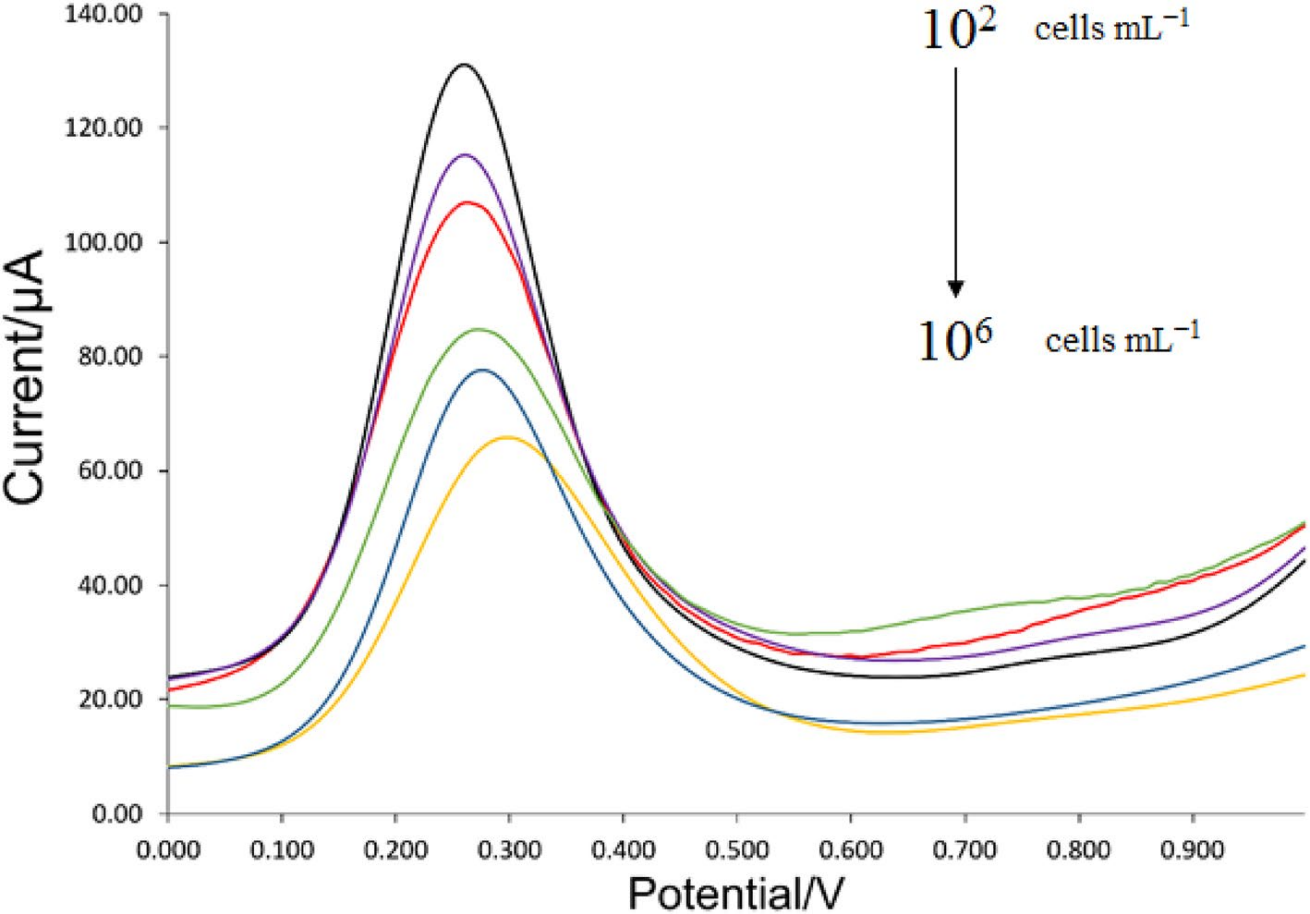
SWV responses of our biosensor for detection of HER2 in different numbers of cells ranging from 10^2^ to 10^6^ cells. All tests were repeated at least 3 times (Temperature of 37 °C; pH 7.4).

**Figure 16:**
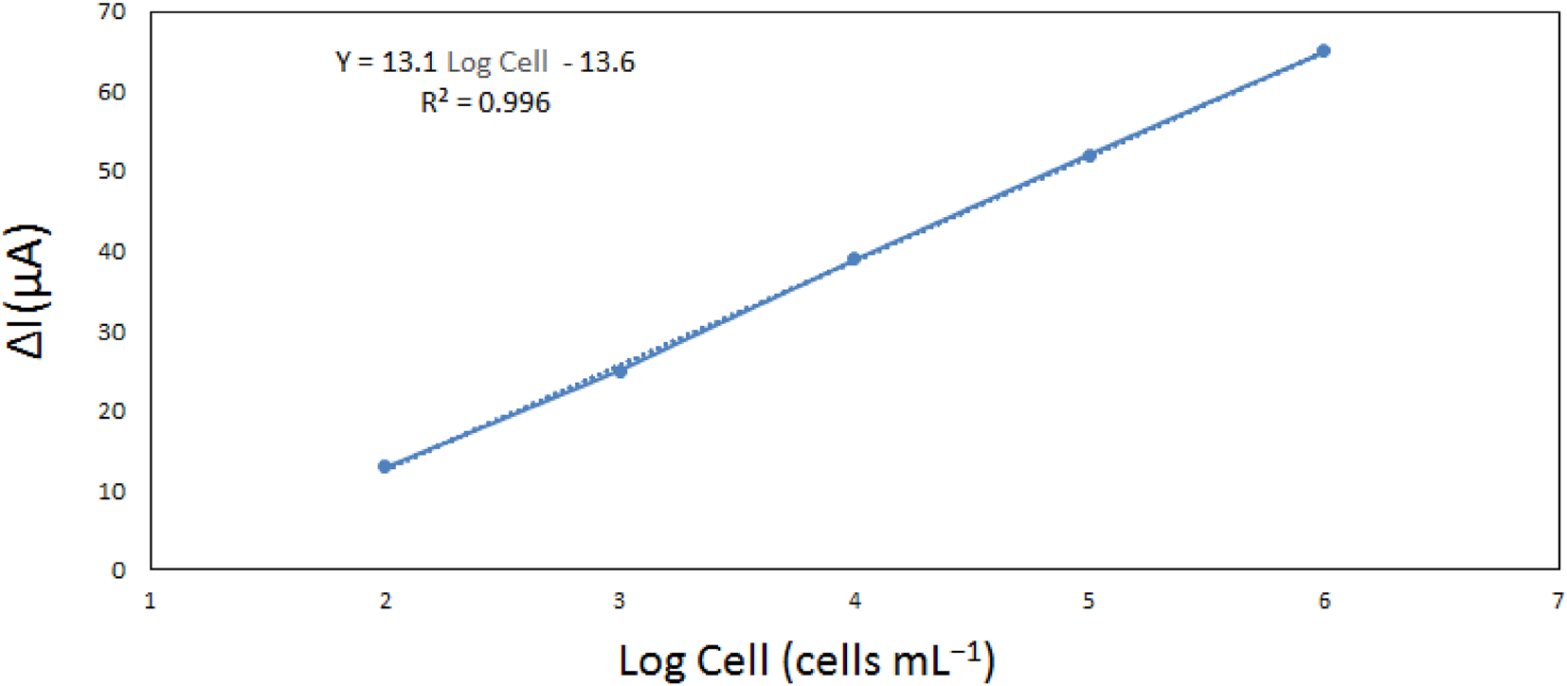
Calibration curve for different numbers of SK-BR3 BC cells under optimal sensing conditions. These tests were performed in a solution containing 5 mM K_3_Fe(CN)_6_, 5 mM K_4_Fe(CN)_6_ and 0.01 M PBS. Every measurement was repeated 3 times (n = 3).

The figure demonstrates how this gadget may linearly identify cancer cells. Since malignant cells are present in low concentration in real-life patient specimens (e.g., blood), the creation of a biosensor for real-time specimen analysis has produced an excellent result, with an R^2^ = 0.996 in the range of 10^2^–10^6^ cells (Figs. 15 and 16).

### Stability

The measurement findings are significantly impacted by the degree of stability. For two weeks, the modified electrode was kept at room temperature to ensure verification. One indicator of the changed electrode's stability is the peak current of the voltammogram. According to the peak current, the amount is 97% of the starting value after one week and 92% of the beginning value after two weeks. Based on the findings, it can be concluded that the changed electrode would be reliable after two weeks.

Furthermore, the analyte is felt using the same process in each of these methods. As previously indicated, biosensing measurements were obtained using SWV, an effective technique. It is possible to conclude that the produced biosensor has superior sensitivity and stability by comparing the response characteristics of the produced biosensor for the detection of HER2 with the response characteristics of other rGO-based biosensors, such as Fe_3_O_4_ and PANI-based biosensors published in the literature (Table 4).

**Table 4:** Different electrochemical biosensors for tumor cell detection.

S. no.	Electrode	Method of immobilization	Electrochemical technique	Detection limit	Detection range	Ref.
1	CoFe_2_O_4_@Ag	citrate	EIS, CV	47 cells mL^−1^	10^2^–10^6^ cells mL^−1^	^37^
2	NFG/AgNPs/PANI	EDC/NHS	EIS, CV	2 cells mL^−1^	10^−5^ × 10^6^ cells mL^−1^	^38^
3	AntiHER2– Fe_3_O_4_ NP bioconjugates	EDC/NHS Cys	EIS, CV	0.995 pg mL^−1^	10–100 ng mL^−1^	^54^
4	Apt-DNA tetrahedral nanostructures	methylene blue	EIS	5 cells mL^−1^	50–1 × 10^6^ cells mL^−1^	^55^
5	AuNPs	thiol aptamer probe sgc8c	EIS	10 cells mL^−1^	10^2^–10^7^ cells mL^−1^	^56^
6	Polymer dot-manganese oxide complexes	–	EIS	3.98 cells mL^−1^	10–10^5^ cells mL^−1^	^57^
7	CeO_2_ at Ir nanorods	EDC/NHS	CV, EIS, DPV	1 cell mL^−1^	2–2 × 10^6^ cells mL^−1^	^58^
8	Nitrogen-doped graphene quantum dots and phytohemagglutinin-L (PHA-L)	-	LSV	2 cells mL^−1^	20–10^6^ cells mL^−1^	^59^
9	Au NCs/MWCNTs-NH_2_	EDC/NHS	CV, EIS, DPV	80 cells mL^−1^	10^2^–10^6^ cells mL^−1^	^60^
10	Present work	EDC/NHS	EIS, CV, SWV	5 cells mL^−1^	10^2^–10^6^ cells mL^−1^	

## Conclusion

Instead of using laborious and backbreaking techniques for early detection, scientists are now required by the medical business and expanding demand for clinical cancer research. To improve survival rates and quality of life, isolating cancer cells is just as crucial as detection. The creation of early-cancer diagnostic systems has been one of the most discussed topics of the last several years. Recent developments in the field of cancer diagnosis demonstrate the significant influence that electrochemical sensing techniques have on the precise, quick, and sensitive identification of different cancer kinds. Specifically, label-free electrochemical biosensors retain key characteristics to provide accurate, economical, and targeted cancer diagnoses that may be used in the future.

This work aimed to create a new electrochemical biosensor for the detection of BC with rGO/Fe_3_O_4_-Nafion/PANI/GCE. Combining rGO with PANI nanosheets might significantly increase biosensor sensitivity and active site attachment. Under ideal circumstances, the integrated EC biosensor can detect as few as 5 cells mL^−1^, and its dynamic range can span up to 100 cells. Outstanding selectivity and sensitivity are offered by the proposed impedimetric biosensor. This electrochemical biosensor has several benefits, such as a high linear dynamic range, excellent repeatability, stability, low detection limit, and no requirement for additional indicators. We thus hope that our research will open new avenues for advancements in modified electrochemical biosensors.

## Data Availability

The datasets used and/or analyzed during the current study available from the corresponding author on reasonable request.

## References

[1] World Health Organization (2023). Global Breast Cancer Initiative Implementation Framework: Assessing, Strengthening and Scaling-up of Services for the Early Detection and Management of Breast Cancer.

[2] Roth GA (2018). Global, regional, and national age-sex-specific mortality for 282 causes of death in 195 countries and territories, 1980–2017: A systematic analysis for the Global Burden of Disease Study 2017. Lancet.

[3] Da Cunha AR (2023). The global, regional, and national burden of adult lip, oral, and pharyngeal cancer in 204 countries and territories: A systematic analysis for the global burden of disease study 2019. JAMA Oncol..

[4] Tian T (2023). Programing immunogenic cell death in breast tumors with designer DNA frameworks. JACS Au.

[5] Gallicchio L, Devasia TP, Tonorezos E, Mollica MA, Mariotto A (2022). Estimation of the number of individuals living with metastatic cancer in the United States. JNCI J. Natl. Cancer Inst..

[6] Amiri FS (2016). Serum tumor markers in chronic kidney disease: As clinical tool in diagnosis, treatment and prognosis of cancers. Renal Fail..

[7] Saito S (2022). Biomarkers of cancer stem cells for experimental research and clinical application. J. Pers. Med..

[8] Conde I, Ribeiro AS, Paredes J (2022). Breast cancer stem cell membrane biomarkers: Therapy targeting and clinical implications. Cells.

[9] Veyssière H, Bidet Y, Penault-Llorca F, Radosevic-Robin N, Durando X (2022). Circulating proteins as predictive and prognostic biomarkers in breast cancer. Clin. Proteom..

[10] Fitzgerald RC, Antoniou AC, Fruk L, Rosenfeld N (2022). The future of early cancer detection. Nat. Med..

[11] Crosby D (2022). Early detection of cancer. Science.

[12] Lawal AT (2023). Recent developments in electrochemical sensors based on graphene for bioanalytical applications. Sens. Bio-Sens. Res..

[13] Bard AJ, Faulkner LR, White HS (2022). Electrochemical Methods: Fundamentals and Applications.

[14] Zare Y, Rhee KY (2022). An innovative model for conductivity of graphene-based system by networked nano-sheets, interphase and tunneling zone. Sci. Rep..

[15] Zare Y, Rhee KY, Park SJ (2022). Advancement of the power-law model and its percolation exponent for the electrical conductivity of a graphene-containing system as a component in the biosensing of breast cancer. Polymers.

[16] Bakhshi A, Jalaly M, Vahedi M (2022). The effect of GO–Fe_3_O_4_ hybrid coating on the magnetic field detection by a tapered optical fiber sensor. Opt. Fiber Technol..

[17] Naghib SM, Behzad F, Rahmanian M, Zare Y, Rhee KY (2020). A highly sensitive biosensor based on methacrylated graphene oxide-grafted polyaniline for ascorbic acid determination. Nanotechnol. Rev..

[18] Ranjan P (2021). Functional ionic liquids decorated carbon hybrid nanomaterials for the electrochemical biosensors. Biosensors.

[19] Peña-Bahamonde J, Nguyen HN, Fanourakis SK, Rodrigues DF (2018). Recent advances in graphene-based biosensor technology with applications in life sciences. J. Nanobiotechnol..

[20] Pandikumar A (2014). Graphene and its nanocomposite material based electrochemical sensor platform for dopamine. RSC Adv..

[21] Yuan W (2013). The edge-and basal-plane-specific electrochemistry of a single-layer graphene sheet. Sci. Rep..

[22] Brownson DAC, Banks CE (2010). Graphene electrochemistry: An overview of potential applications. Analyst.

[23] Yang J, Hu P, Yu G (2019). Perspective of graphene-based electronic devices: Graphene synthesis and diverse applications. APL Mater..

[24] Pei S, Cheng H-M (2012). The reduction of graphene oxide. Carbon.

[25] Gao W (2015). Graphene Oxide: Reduction Recipes, Spectroscopy, and Applications.

[26] Raza H (2012). Graphene Nanoelectronics: Metrology, Synthesis, Properties and Applications.

[27] Lee XJ (2019). Review on graphene and its derivatives: Synthesis methods and potential industrial implementation. J. Taiwan Inst. Chem. Eng..

[28] Tewatia K, Sharma A, Sharma M, Kumar A (2021). Synthesis of graphene oxide and its reduction by green reducing agent. Mater. Today Proc..

[29] Khosroshahi Z, Kharaziha M, Karimzadeh F, Allafchian A (2018). Green reduction of graphene oxide by ascorbic acid. AIP Conf. Proc..

[30] Kandasamy G, Maity D (2015). Recent advances in superparamagnetic iron oxide nanoparticles (SPIONs) for in vitro and in vivo cancer nanotheranostics. Int. J. Pharm..

[31] Xu J-K (2014). Bio and nanomaterials based on Fe_3_O_4_. Molecules.

[32] Xianyu Y, Wang Q, Chen Y (2018). Magnetic particles-enabled biosensors for point-of-care testing. TrAC Trends Anal. Chem..

[33] Lu B-W, Chen W-C (2006). A disposable glucose biosensor based on drop-coating of screen-printed carbon electrodes with magnetic nanoparticles. J. Magn. Magn. Mater..

[34] Goswami S, Nandy S, Fortunato E, Martins R (2023). Polyaniline and its composites engineering: A class of multifunctional smart energy materials. J. Solid State Chem..

[35] Prenzel N, Fischer OM, Streit S, Hart S, Ullrich A (2001). The epidermal growth factor receptor family as a central element for cellular signal transduction and diversification. Endocr. Relat. Cancer.

[36] Yarden Y, Sliwkowski MX (2001). Untangling the ErbB signalling network. Nat. Rev. Mol. Cell Biol..

[37] Vajhadin F (2022). MXene-based cytosensor for the detection of HER2-positive cancer cells using CoFe_2_O_4_@Ag magnetic nanohybrids conjugated to the HB5 aptamer. Biosens. Bioelectron..

[38] Salahandish R (2018). Nano-biosensor for highly sensitive detection of HER2 positive breast cancer. Biosens. Bioelectron..

[39] Jensen WA (2017). Response surface methodology: Process and product optimization using designed experiments. J. Qual. Technol..

[40] Ankenman BE, Dean AM (2003). Quality improvement and robustness via design of experiments. Handb. Stat..

[41] Zhu X (2012). Reduction of graphene oxide via ascorbic acid and its application for simultaneous detection of dopamine and ascorbic acid. Int. J. Electrochem. Sci.

[42] Zong P (2013). Synthesis and application of magnetic graphene/iron oxides composite for the removal of U(VI) from aqueous solutions. Chem. Eng. J..

[43] Wang C (2011). Preparation of a graphene-based magnetic nanocomposite for the removal of an organic dye from aqueous solution. Chem. Eng. J..

[44] Darowicki K, Kawula J (2004). Impedance characterization of the process of polyaniline first redox transformation after aniline electropolymerization. Electrochim. Acta.

[45] Wang Y, Shi Z, Yin J (2011). Facile synthesis of soluble graphene via a green reduction of graphene oxide in tea solution and its biocomposites. ACS Appl. Mater. Interfaces.

[46] Kurniasari (2017). Defect and magnetic properties of reduced graphene oxide prepared from old coconut shell. IOP Conf. Ser. Mater. Sci. Eng..

[47] Avinash MB, Subrahmanyam KS, Sundarayya Y, Govindaraju T (2010). Covalent modification and exfoliation of graphene oxide using ferrocene. Nanoscale.

[48] Wang L, Huang Y, Li C, Chen J, Sun X (2015). Hierarchical composites of polyaniline nanorod arrays covalently-grafted on the surfaces of graphene@ Fe_3_O_4_@ C with high microwave absorption performance. Compos. Sci. Technol..

[49] Mathew, J., Sathishkumar, M., Kothurkar, N. K., Senthilkumar, R. & Sabarish Narayanan, B. in *IOP Conference Series: Materials Science and Engineering.* 012138–012138 (IOP Publishing, 2023).

[50] Mondal S, Rana U, Malik S (2017). Reduced graphene oxide/Fe_3_O_4_/polyaniline nanostructures as electrode materials for an all-solid-state hybrid supercapacitor. J. Phys. Chem. C.

[51] Xie W (2022). Magnetoresistive and piezoresistive polyaniline nanoarrays in-situ polymerized surrounding magnetic graphene aerogel. Adv. Compos. Hybrid Mater..

[52] Khan MAM, Khan W, Ahamed M, Alhazaa AN (2019). Investigation on the structure and physical properties of Fe_3_O_4_/RGO nanocomposites and their photocatalytic application. Mater. Sci. Semicond. Process..

[53] Manna K, Srivastava SK (2017). Fe_3_O_4_@ carbon@ polyaniline trilaminar core–shell composites as superior microwave absorber in shielding of electromagnetic pollution. ACS Sustain. Chem. Eng..

[54] Emami M, Shamsipur M, Saber R, Irajirad R (2014). An electrochemical immunosensor for detection of a breast cancer biomarker based on antiHER2–iron oxide nanoparticle bioconjugates. Analyst.

[55] Yang L, Yin X, Gai P, Li F (2020). A label-free homogeneous electrochemical cytosensor for the ultrasensitive detection of cancer cells based on multiaptamer-functionalized DNA tetrahedral nanostructures. Chem. Commun..

[56] Wang S-S (2019). Direct plasmon-enhanced electrochemistry for enabling ultrasensitive and label-free detection of circulating tumor cells in blood. Anal. Chem..

[57] Won HJ (2020). Wireless label-free electrochemical detection of cancer cells by MnO_2_-decorated polymer dots. Sens. Actuators B Chem..

[58] Shen H (2020). Ultrasensitive aptasensor for isolation and detection of circulating tumor cells based on CeO_2_@ Ir nanorods and DNA walker. Biosens. Bioelectron..

[59] Tran HL, Dega NK, Lu S-M, Huang Y-F, Doong R-A (2022). Ultrasensitive detection of breast cancer cells with a lectin-based electrochemical sensor using N-doped graphene quantum dots as the sensing probe. Sens. Actuators B Chem..

[60] Yang Y, Fu Y, Su H, Mao L, Chen M (2018). Sensitive detection of MCF-7 human breast cancer cells by using a novel DNA-labeled sandwich electrochemical biosensor. Biosens. Bioelectron..

